# The effect of different preoperative left atrial appendage emptying speeds on left atrial function in patients with persistent atrial fibrillation after left atrial appendage closure combined with catheter ablation

**DOI:** 10.1186/s12872-022-02842-z

**Published:** 2022-09-06

**Authors:** Chao Yang, Jing Yang, Qian Liu, Ling You, Jinglan Wu, Yanan Zhang, Lianxia Wang, Ruiqin Xie

**Affiliations:** Department of Cardiology, The Second Hospital of Hebei Medical University, No. 125 of Heping West Road Road, Xinhua District, Shijiazhuang, Hebei People’s Republic of China

**Keywords:** Left atrial appendage closure, Catheter ablation, Left atrial function, Left atrial appendage emptying velocity, Spot tracking echocardiography

## Abstract

**Objective:**

The present study aimed to investigate whether different preoperative left atrial appendage emptying speeds (LAAEVs) have an effect on left atrial function in patients with sinus arrhythmia after left atrial appendage closure (LAAC) combined with catheter ablation (CA).

**Methods:**

A total of 163 patients with persistent non-valvular atrial fibrillation who received combined LAAC+CA surgery were included in the present study. Regular follow-up was conducted for 12 months, and patients with complete data and successful sinus rhythm recovery were selected as the study subjects (*n* = 82). The patients were divided into two groups: the LAAEV < 25 cm/s group and the LAAEV ≥ 25 cm/s group (*n* = 41 each). The propensity score was used for matching according to gender, age, CHA_2_DS_2_-VASc score, and HAS-BLED score. The changes in the two groups in LA structure, storage function, conduit function, and pump function were compared.

**Results:**

Before surgery, the maximum LA volume (LAV_max_) and minimum LA volume (LAV_min_) were greater in the LAAEV < 25 cm/s group than in the LAAEV ≥ 25 cm/s group. The LA storage function (eg. Ƹ and SRs), conduit function (eg. SRe), and pump function (eg. SRa) were all worse in the LAAEV < 25 cm/s group than in the LAAEV ≥ 25 cm/s group. After the combined LAAC+CA surgery, the LA storage, conduit, and pump functions improved in both groups. At 12 months after surgery, there were no statistically significant differences between the two groups.

**Conclusion:**

Before combined LAAC+CA surgery, the LA structure and function of the LAAEV < 25 cm/s group were worse than those of the LAAEV ≥ 25 cm/s group. However, after LAAC+CA surgery, the LA structure and function of the patients were improved, and there were no significant differences between the two groups. Inferred improvement in LA structure and function in the LAAEV < 25 cm/s group was superior to that in the LAAEV ≥ 25 cm/s group.

## Introduction

Atrial fibrillation (AF) is a serious threat to human health that causes stroke and thromboembolic diseases and aggravates the symptoms of heart failure [1]. The latest research confirms that almost 100% of AF-induced thrombus cases originate from the left atrial (LA) appendage (LAA) [2]. Compared with warfarin, LAA closure (LAAC) can significantly reduce the incidence of hemorrhagic stroke, disabling/fatal stroke, and cardiovascular and all-cause death [3]. The use of catheter ablation (CA) combined with LAAC for sinus rhythm restoration can alleviate the symptoms of heart failure and prevent the occurrence of cardiogenic thromboembolic events. It can also further reduce bleeding events caused by the long-term use of anticoagulant drugs. Multi-center studies have confirmed the safety and effectiveness of LAAC+CA in the treatment of AF [4, 5].

Studies have also shown that, as well as restoring the sinus rhythm in patients with persistent AF, CA leads to a significant reduction in postoperative LA volume and improves LA function [6, 7]. It has therefore been suggested that CA positively affects the structure and function of the LA. As a part of the LA, the LAA has a specific capacity. Compared with the LA, the LAA has greater active diastolic and contraction functions, which significantly impacts LA compliance and function [8].

Although results regarding the impact of LAAC on the structure and function of the LA are controversial [9–12], previous research conducted by the Second Hospital of Hebei Medical University confirmed an improvement in LA structure and function after LAAC+CA as well as restoration of the sinus rhythm in patients with persistent AF. The research also found, however, that this improvement was primarily caused by CA: the additional LAAC program did not improve LA structure and function after CA [7]. However, there is currently no research on the effect of LAA emptying speed (LAAEV) on the structure and function of the LA after LAAC+CA. The present study therefore compared the changes in LA structure and function between two groups after LAAC+CA in order to analyze the impact of LAAEV on postoperative LA storage, conduit function, and pump function.

## Methods

### Study population

Patients with persistent non-valvular AF who received LAAC+CA treatment in the First Department of Cardiology at the Second Hospital of Hebei Medical University between January 2017 and September 2020 were enrolled as the study subjects. Persistent AF was defined as lasting > 7 days, and long-term persistent AF was defined as lasting > 12 months.

Inclusion criteria: (1) Patients with a high stroke risk (CHA_2_DS_2_-VASc score ≥ 2 in male patients and ≥ 3 in female patients); (2) patients aged > 18 years and < 80 years; (3) patients who met one of the following criteria: the patient was not suitable for long-term standard anticoagulation therapy, the patient was receiving standard anticoagulant therapy based on coagulation therapy but still suffered an embolism, or the patient had a HAS-BLED score of ≥ 3.

Exclusion criteria: (1) Patients with an inner left atrium diameter > 55 mm; (2) patients with transesophageal echocardiography (TEE)-detected LAA thrombosis; (3) patients with severe mitral valve disease or sizeable pericardial effusion; (4) patients with coagulation dysfunction; (5) patients with combined hyperthyroidism; (6) patients with a life expectancy ≤ 1 year.

**Fig. 1:**
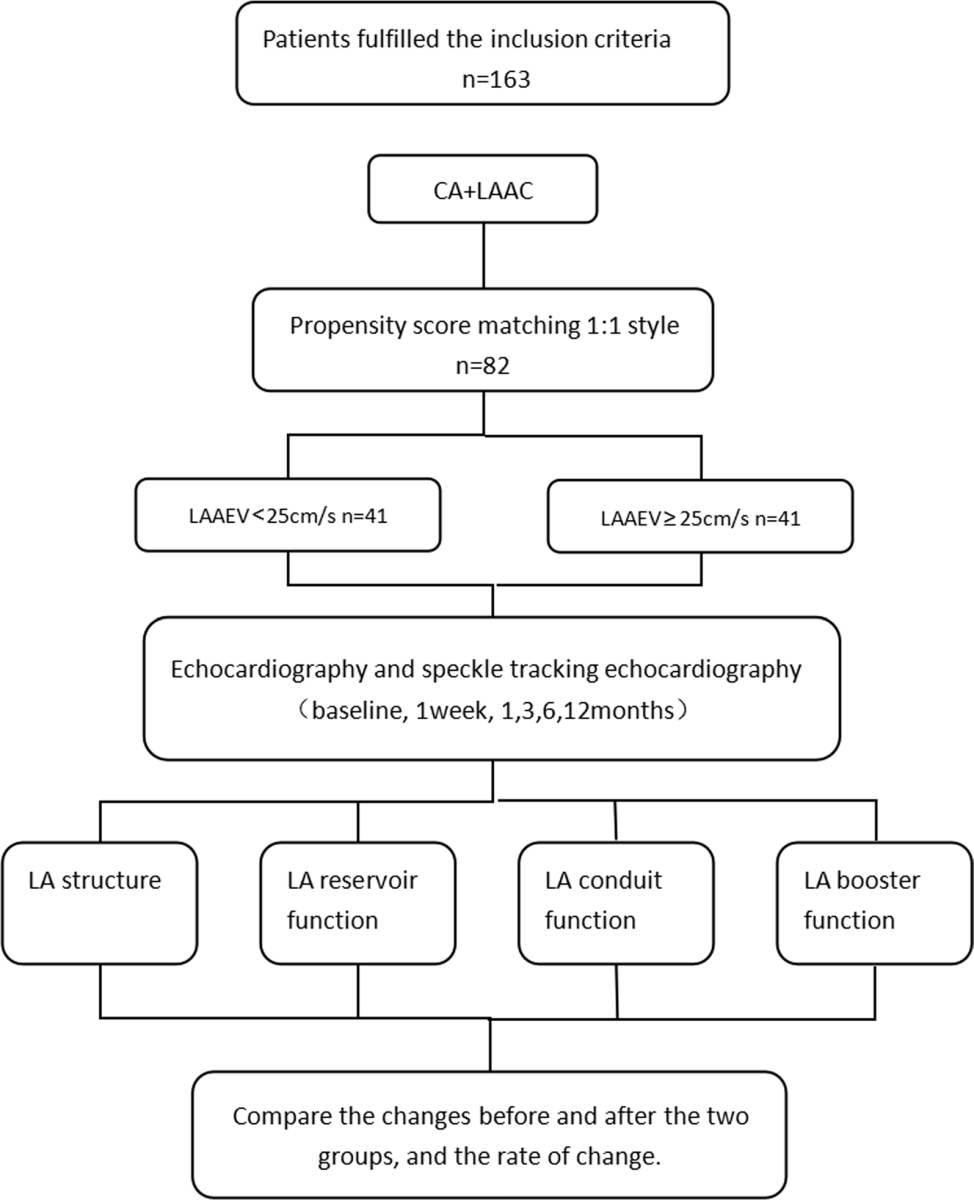
Flowchart of the study procedure. LA, left atrial; LAAC, left atrial appendage closure; CA, catheter ablation; LAAEV Left atrial appendage emptying velocity

The experimental process is shown in Fig. 1. This study was approved by the Ethics Committee of the Second Hospital of Hebei Medical University.

### Surgical procedure

All operations were performed under local anesthesia. Before surgery, all patients (excluding patients with LA thrombosis) underwent TEE, and the LAAEV was recorded. Under the guidance of the CARTO 3 (Biosense Webster, USA) system, a Thermocool SmartTouch catheter (Biosense Webster, USA) was used to isolate the pulmonary veins, and a Lasso catheter (Biosense Webster, USA) was used to verify the bidirectional isolation of all pulmonary veins. No additional ablation lines were performed in this study. The sinus rhythm of all patients was recovered through ablation, medication, or electrical cardioversion. After ablation, blocking was conducted according to routine procedure. Watchman devices (Boston Scientific, Marlborough, Massachusetts, USA) were used as implanted occluders, and the PASS principle was followed to release the occluders.

### Echocardiography

Before surgery and at 1 week and 1, 3, 6, and 12 months after the procedure, the same experienced echocardiography specialist used the same ultrasound system (iE33 machine with X3 probe; Eindho, Philips Medical Systems, Netherlands) to test each patient’s AF function. The left atrium diameter (LAD) at the end of the systole was measured on the parasternal long-axis view. The area-length method was used in the apical four-chamber part.

The maximum LA volume (LAV_max_) of the left ventricle at the end of systole and the minimum LA volume (LAV_min_) of the left ventricle at the end of diastole were measured. The CMQ (QLAB Ultrasound Cardiac Analysis; Philips Medical Systems, Netherlands) mode was started, the apical four-chamber view was obtained, three cardiac cycles were manually tracked, a stable strain rate curve was obtained, the LA full volume image was selected, and the LA sampling point was placed. The strain rate curves of the four chambers on the septal side, sidewall, and top of the atrium were obtained.

**Fig. 2:**
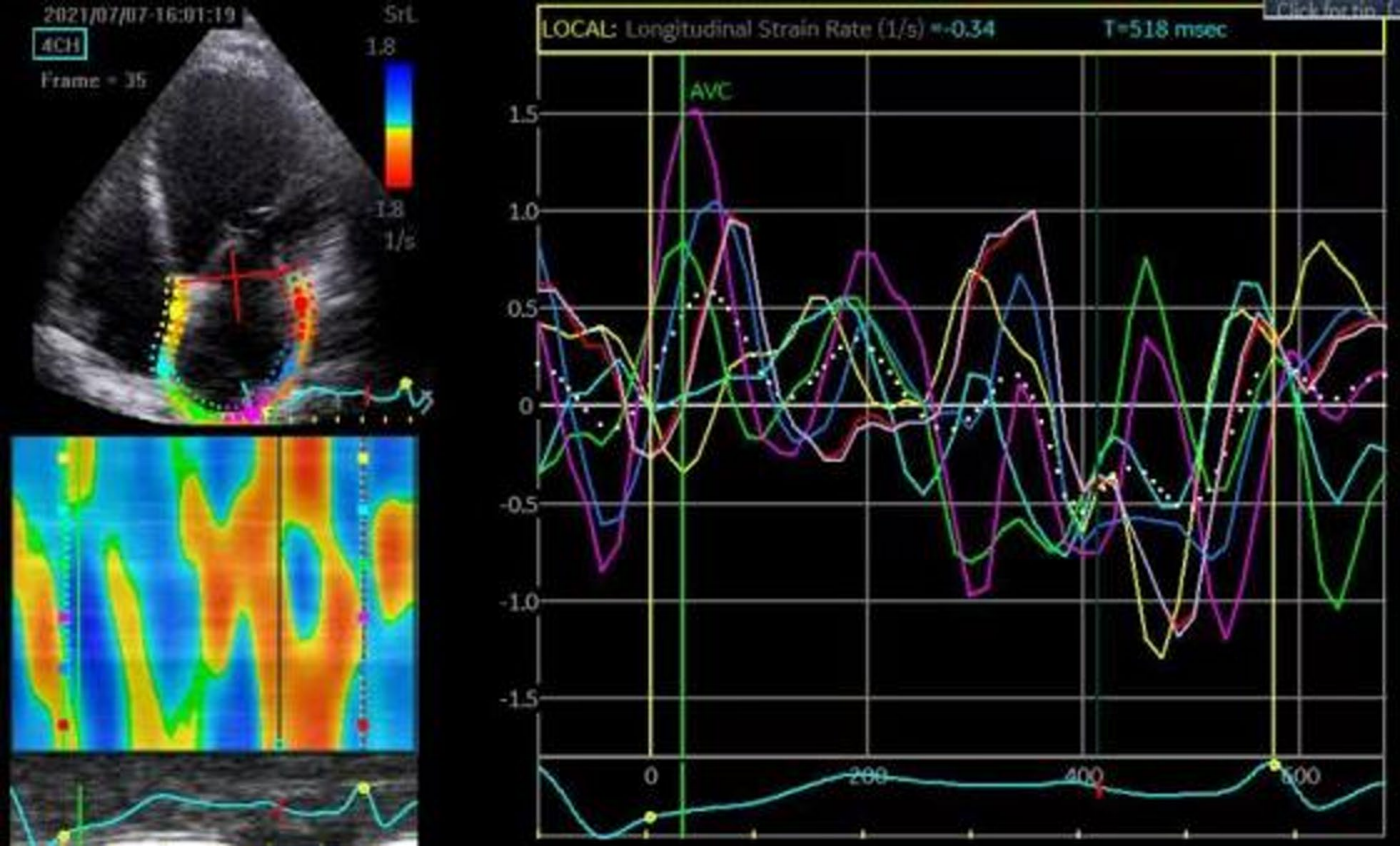
Measurement of LA strain and strain rate in apical four chamber view

Previous research conducted at the Second Hospital of Hebei Medical University confirmed this measurement method [13], which can be used to determine the total systolic strain and the SR, reflecting the storage function of the left atrium. The early diastolic early diastolic peak strain rate (SRe) reflects LA conduit function, and the end-diastolic atrial systole peak strain rate (SRa) indicates LA pumping function (see Fig. 2).

### Postoperative follow-up and assessment of heart rhythm

All patients received routine oral antiarrhythmic and anticoagulant drugs after surgery. Three months later, TEE was performed to exclude device-related thrombosis and a residual shunt of ≥ 5 mm; the anticoagulant drugs were then discontinued and replaced with antiplatelet drugs (aspirin 100 mg, once a day, and clopidogrel 75 mg, once a day) for 3 months, after which the patients took only aspirin for a year. A Holter examination was performed at 3, 6, and 12 months after surgery; AF or an atrial flutter duration of > 30 s was regarded as a recurrence of AF, and these patients were excluded from the scope of the study.

### Statistical analysis

The SPSS software version 22 (IBM, Armonk, NY, USA) was used for data analysis, and Kolmogorov–Smirnov test (K–S) was applied to test whether the data conformed to normal distribution. Normally distributed data were expressed as mean ± standard deviation, and non-normally distributed data were expressed as interquartile range. The count data were expressed as a percentage (%).

A student’s *t*-test (for normally distributed data) or a Mann–Whitney U test (for non-normally distributed data) and a χ^2^ test were used to compare the baseline parameters of the two patient groups. The LA structure and function in the two groups were compared using repeated-measures analysis of variance. The degrees of change in LA structure and function were expressed as the rate of change (observation time value − preoperative value/preoperative value), and these rates of change in LA structure and function in the two groups were compared using a repeated-measures analysis of variance. P < 0.05 was considered statistically significant.

## Results

### Baseline data

A total of 163 patients with persistent non-valvular AF who underwent LAAC+CA were included in the present study. Regular follow-up was conducted for 12 months, and patients with complete data and successful sinus rhythm recovery were selected as the study subjects. Among the 163 patients that were initially included, 82 met the study requirements and were divided into two groups according to LAAEV [14]: the LAAEV < 25 cm/s group and the LAAEV ≥ 25 cm/s group (*n* = 41 each). The basic characteristics of the two patient groups are shown in Table 1. There were no significant differences between the two groups in age, gender, medical history, proportion of long-term persistent AF, CHA_2_DS_2_-VASc score, and HAS-BLED score (P > 0.05), indicating that the basic clinical data of the two groups were comparable.

**Table 1:** Baseline characteristics of the population

	LAAEV < 25 cm/s	LAAEV ≥ 25 cm/s	*P*-value
Age (years)	62.59 ± 6.83	62.98 ± 6.51	0.97
Sex male *n* (%)	26 (63)	22 (54)	0.37
Type of atrial fibrillation			0.18
Persistent AF *n* (%)	21 (51)	27 (66)	
Long-standing persistent AF *n* (%)	20 (49)	14 (34)	
Smoke *n* (%)	6 (15)	5 (12)	0.75
Alcohol *n* (%)	9 (22)	4 (10)	0.13
Labile INR *n* (%)	2 (5)	4 (10)	0.40
Hypertension *n* (%)	24 (59)	27 (66)	0.49
Coronary artery disease *n* (%)	15 (37)	15 (37)	1
Diabetes mellitus *n* (%)	11 (27)	6 (15)	0.17
Congestive heart failure	21 (51)	24 (59)	0.51
Stroke *n* (%)	19 (46)	22 (54)	0.51
Bleeding *n* (%)	3 (7)	6 (15)	0.29
CHA2DS2- VASc score	3 (3, 5)	3 (2, 3)	0.42
HAS-BLED score	4 (3, 5)	3 (2, 3)	0.55

Data are shown as mean ± SD or n (%)

### Left atrium structure and function assessment

#### Left atrium structure (LAD, LAV_max_, and LAV_min_)

There were no significant differences in LAD before and after treatment in the LAAEV < 25 cm/s group or the LAAEV ≥ 25 cm/s group. The preoperative LAV_max_ in the LAAEV < 25 cm/s group was lower than in the LAAEV ≥ 25 cm/s group (91.46 ± 16.17 vs. 81.49 ± 14.28; P = 0.004). The LAV_max_ in both groups was decreased after surgery compared with before surgery, but from 1 month after surgery, there was no statistically significant difference between the two groups (78.80 ± 18.22 vs. 72.85 ± 15.00; P = 0.11). The 3 months rate of change (ΔLAV_max_ = [LAV_3months_ − LAV_baseline_]/LAV_baseline_) (− 0.18 ± 0.17 vs. − 0.08 ± 0.14; P = 0.004), 6 months rate of change (ΔLAV_max_ = [LAV_6months_ − LAV_baseline_]/LAV_baseline_) (− 0.181 ± 0.14 vs. − 0.11 ± 0.11; P = 0.003), and 12 months rate of change (∆LAV_max_ = [LAV_12months_ − LAV_baseline_]/LAV_baseline_) (− 0.20 ± 0.16 vs. − 0.14 ± 0.11; P = 0.036) were statistically significant. The preoperative LAV_min_ was lower in the LAAEV < 25 cm/s group than in the LAAEV ≥ 25 cm/s group (65.85 ± 18.17 vs. 56.02 ± 16.21; P = 0.012).

**Fig. 3:**
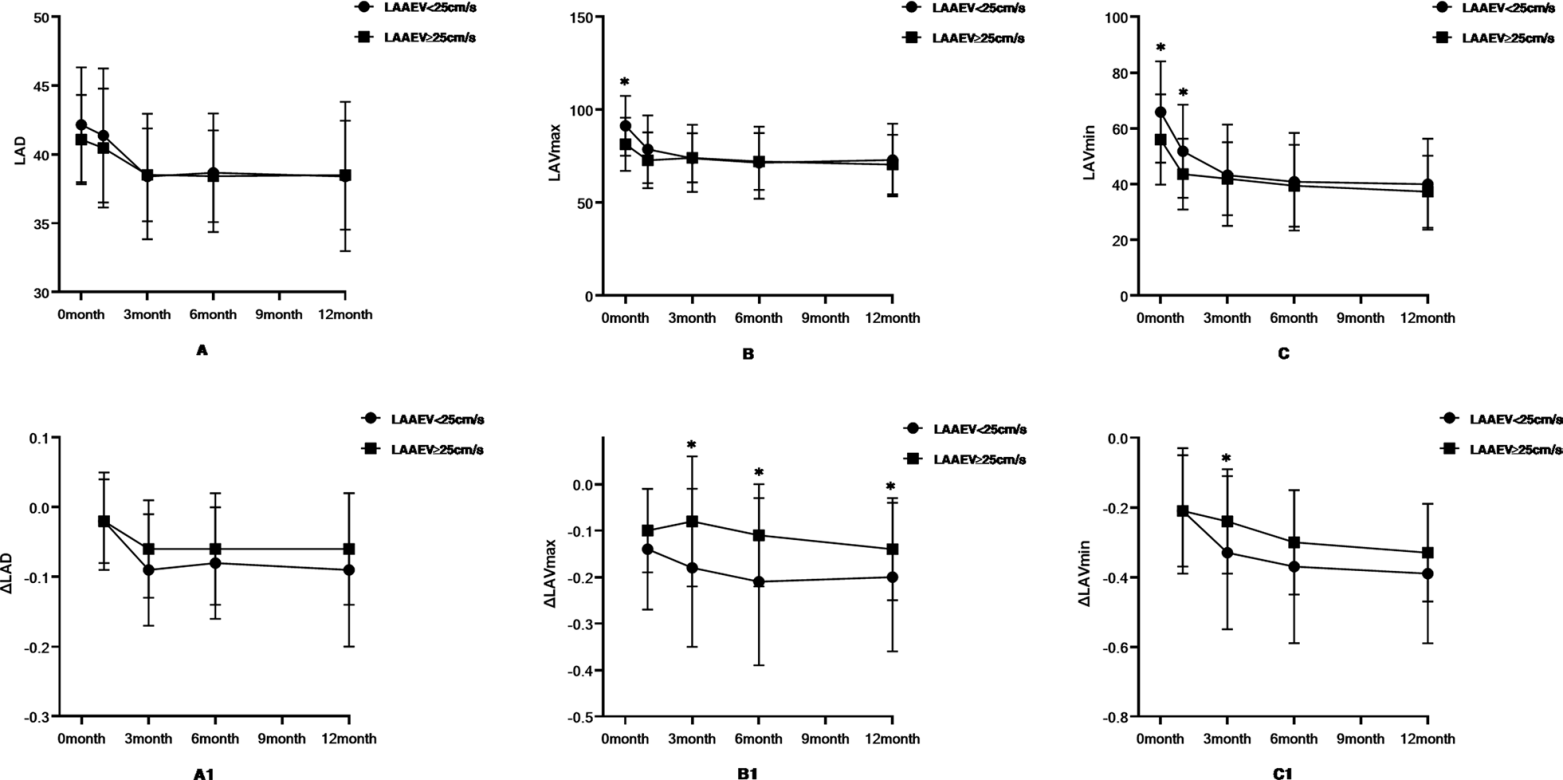
Comparisons of the LA diameter and LA volumes between the LAAEV < 25 cm/s and LAAEV ≥ 25 cm/s group. **A** Comparison of LAD between the two groups. **A1** Comparison of the rate of change of LAD between the two groups (**B**) comparison of LAV_max_ between the two groups. **B1** Comparison of the rate of change of LAV_max_ between the two groups (**C**) comparison of LAV_min_ between the two groups. **C1** Comparison of the rate of change of LAV_min_ between the two groups. Variables are expressed as means ± standard deviations. *P < 0.05 LAD, left atrial diameter; LAV_max_, maximum volume of left atrium; LAV_min_, Minimum volume of left atrium

The LAV_min_ in both groups was decreased after surgery compared with before surgery, but from 3 months after surgery, there was no statistically significant difference between the two groups (43.24 ± 18.18 vs. 41.93 ± 13.10; P = 0.708). The 3 months rate of change (ΔLAV_min_ = [LAV_3months_ − LAV_baseline_]/LAV_baseline_) (− 0.33 ± 0.22 vs. − 0.24 ± 0.15; P = 0.033) in the two groups was statistically significant (see Tables 2 and 3 and Fig. 3).

**Table 2:** Evolution of LA structure and function between LAAEV < 25 cm/s group and LAAEV ≥ 25 cm/s group

	Baseline	1 Week	1 Month	3 Months	6 Months	12 Months	*F* value	*P*-value
*LAAEV* < *25 cm/sgroup(n* = *41)*								
LAD	42.15 ± 4.17		41.37 ± 4.86	38.39 ± 4.56	38.66 ± 4.31	38.39 ± 5.42	14.41	< 0.001
LAV_max_	91.46 ± 16.17		78.80 ± 18.22	73.95 ± 18.12	71.56 ± 19.37	73.02 ± 19.54	14.16	< 0.001
LAV_min_	65.85 ± 18.17		51.80 ± 16.72	43.24 ± 18.18	40.85 ± 17.55	39.98 ± 16.33	31.49	< 0.001
Ƹ (4-chamber)	14.22 ± 4.26	–	19.56 ± 5.77	24.39 ± 7.06	27.17 ± 8.02	27.66 ± 6.44	50.06	< 0.001
SRs (4-chamber)	0.92 ± 0.26	–	1.07 ± 0.32	1.32 ± 0.33	1.47 ± 0.32	1.51 ± 0.36	34.87	< 0.001
SRe (4-chamber)	− 1.32 ± 0.61	-	− 1.36 ± 0.33	− 1.42 ± 0.43	− 1.54 ± 0.48	− 1.63 ± 0.47	3.85	0.01
SRa (4-chamber)	–	− 1.05 ± 0.48	− 1.30 ± 0.55	− 1.46 ± 0.52	− 1.73 ± 0.69	− 1.82 ± 0.58	34.13	< 0.001
*LAAEV* ≥ *25 cm/s group (n* = *41)*								
LAD	41.07 ± 3.24		40.46 ± 4.31	38.51 ± 3.37	38.41 ± 3.33	38.49 ± 3.95	8.01	< 0.001
LAV_max_	81.49 ± 14.28		72.85 ± 15.00	74.20 ± 13.20	72.17 ± 15.28	70.59 ± 16.06	16.13	< 0.001
LAV_min_	56.02 ± 16.21		43.61 ± 12.71	41.93 ± 13.10	39.41 ± 14.67	37.32 ± 12.96	44.58	< 0.001
Ƹ (4-chamber)	19.51 ± 7.78	–	23.95 ± 7.52	29.39 ± 8.13	30.29 ± 7.41	30.73 ± 8.74	23.90	< 0.001
SRs (4-chamber)	1.15 ± 0.33	–	1.22 ± 0.31	1.45 ± 0.38	1.62 ± 0.38	1.68 ± 0.48	16.42	< 0.001
SRe (4-chamber)	− 1.64 ± 0.52	–	− 1.52 ± 0.40	− 1.62 ± 0.43	− 1.79 ± 0.46	− 1.83 ± 0.51	9.89	< 0.001
SRa (4-chamber)	–	− 1.35 ± 0.48	− 1.63 ± 0.52	− 1.74 ± 0.54	− 1.98 ± 0.59	− 1.99 ± 0.56	18.86	< 0.001

Data are shown as mean ± SD

**Table 3:** Evolution of the rate of change of LA structure and function between LAAEV < 25 cm/s group and LAAEV ≥ 25 cm/s group

	1 month–1 week	1 Month-baseline	3 Months-baseline	6 Months-baseline	12 Months-baseliane	F value	P-value
*LAAEV* < *25 cm/sgroup(n* = *41)*							
LAD		− 0.02 ± 0.06	− 0.09 ± 0.08	− 0.08 ± 0.08	− 0.09 ± 0.11	11.11	< 0.001
LAV_max_		− 0.14 ± 0.13	− 0.18 ± 0.17	− 0.21 ± 0.18	− 0.20 ± 0.16	4.94	0.005
LAV_min_	− 0.21 ± 0.18		− 0.33 ± 0.22	− 0.37 ± 0.22	− 0.39 ± 0.20	19.11	< 0.001
Ƹ (4-chamber)	–	0.45 ± 0.49	0.84 ± 0.70	1.06 ± 0.81	1.09 ± 0.69	15.64	< 0.001
SRs (4-chamber)	–	0.24 ± 0.45	0.56 ± 0.67	0.71 ± 0.53	0.75 ± 0.58	30.02	< 0.001
SRe (4-chamber)	–	0.02 ± 0.55	0.08 ± 0.67	0.15 ± 0.63	0.23 ± 0.75	2.89	0.048
SRa (4-chamber)	0.30 ± 0.39		0.49 ± 0.57	0.78 ± 0.75	0.92 ± 0.75	15.65	< 0.001
*LAAEV* ≥ *25 cm/s group (n* = *41)*							
LAD		− 0.02 ± 0.07	− 0.06 ± 0.07	− 0.06 ± 0.08	− 0.06 ± 0.08	4.81	0.006
LAV_max_		− 0.10 ± 0.09	− 0.08 ± 0.14	− 0.11 ± 0.11	− 0.14 ± 0.11	3.45	0.026
LAV_min_	− 0.21 ± 0.16		− 0.24 ± 0.15	− 0.30 ± 0.15	− 0.33 ± 0.14	14.43	< 0.001
Ƹ (4-chamber)	–	0.33 ± 0.48	0.67 ± 0.67	0.73 ± 0.62	0.71 ± 0.53	15.20	< 0.001
SRs (4-chamber)	–	0.16 ± 0.47	0.45 ± 0.77	0.53 ± 0.47	0.58 ± 0.61	21.11	< 0.001
SRe (4-chamber)	–	− 0.02 ± 0.31	0.05 ± 0.32	0.13 ± 0.26	0.17 ± 0.30	6.41	0.001
SRa (4-chamber)	0.24 ± 0.28		0.35 ± 0.45	0.64 ± 0.46	0.56 ± 0.52	7.95	< 0.001

Data are shown as mean ± SD

#### Left atrium storage function (Ƹ and SRs)

The preoperative four-chamber Ƹ and SRs were lower in the LAAEV < 25 cm/s group (14.22 ± 4.26 vs. 19.51 ± 7.78; P = 0.001) than in the LAAEV ≥ 25 cm/s group (0.92 ± 0.26 vs. 1.15 ± 0.44; P = 0.004). After surgery, Ƹ and SRs increased in both groups. At 6 months after surgery, there was no significant difference between the two groups (27.17 ± 8.02 vs. 30.29 ± 7.41; P = 0.071). The 6 months rate of change (∆Ƹ = [Ƹ6months − Ƹbaseline]/Ƹbaseline) (1.06 ± 0.81 vs. 0.73 ± 0.62; P = 0.037) and 12-months rate of change (∆Ƹ = [Ƹ12months − Ƹbaseline]/Ƹbaseline) (1.09 ± 0.69 vs. 0.71 ± 0.53; P = 0.007) were statistically significant.

At 3 months after surgery, there was no statistically significant difference between the two groups in the four-chamber SRs (1.32 ± 0.33 vs. 1.45 ± 0.38; P = 0.113). There was also no statistical difference in the rate of change between the two groups, but there was a statistical trend.

#### Left atrial conduit function (SRe)

The preoperative SRe was lower in the LAAEV < 25 cm/s group than in the LAAEV ≥ 25 cm/s group (− 1.32 ± 0.61 vs. − 1.64 ± 0.52; P = 0.012), and the SRes in both groups after surgery were increased compared with before surgery. There was no statistical difference between the two groups at 12 months after surgery (− 1.63 ± 0.47 vs. − 1.83 ± 0.51; P = 0.062). The rates of change in the two groups were not statistically significant, but there was a statistical trend.

#### Left atrial pump function (SRa)

**Fig. 4:**
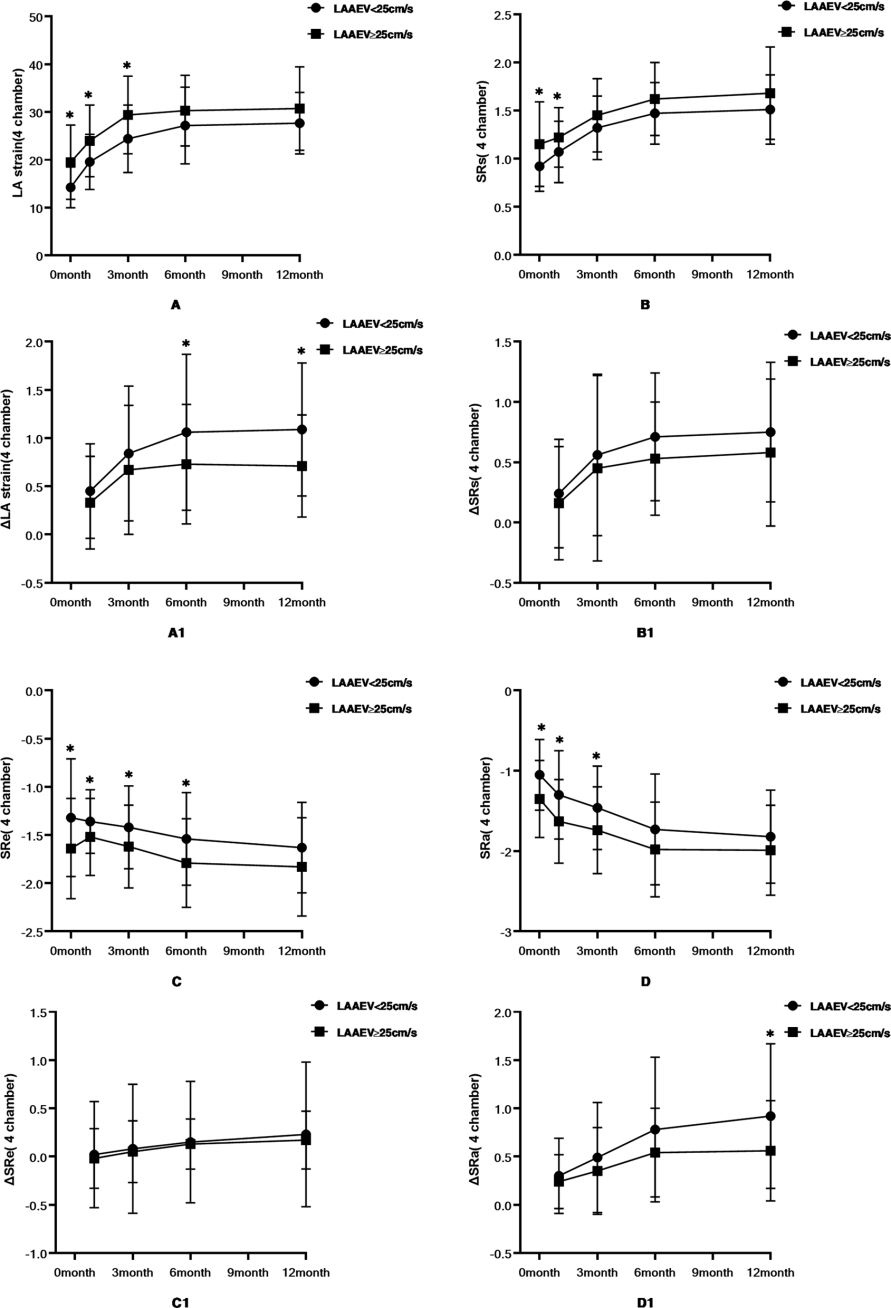
Comparison of left atrial function between the two groups as assessed by 4 chambers strain indices, (**A**) comparison of LA strain between the two groups. **A1** Comparison of the rate of change of LA strain between the two groups (**B**) comparison of SRs between the two groups. **B1** Comparison of the rate of change of SRs between the two groups (**C**) comparison of SRe between the two groups. **C1** Comparison of the rate of change of SRe between the two groups. **D** Comparison of SRa between the two groups. **D1** Comparison of the rate of change of SRa between the two groups. Variables are expressed as mean ± standard deviation. *P < 0.05 SRs, strain rate during ventricular contraction; SRe, ventricular strain rate during early diastole; SRa, atrial strain rate

The preoperative SRa was lower in the LAAEV < 25 cm/s group than in the LAAEV ≥ 25 cm/s group (− 1.05 ± 0.44 vs. − 1.35 ± 0.48; P = 0.005), and the SRa increased in both groups after surgery. At 6 months after surgery, there was no statistical difference between the two groups (− 1.73 ± 0.69 vs. − 1.98 ± 0.59; P = 0.09). The rates of change in the two groups at 12 months (∆SRa = [SRa12 months − SRabaseline]/SRabaseline) (0.92 ± 0.75 vs. 0.56 ± 0.52; P = 0.015) were statistically significant (see Tables 2 and 3 and Fig. 4).

### Clinical outcomes

For the 82 patients that were eventually included in the study, propensity score matching was used according to gender, age, CHA_2_DS_2_-VASc score, and HAS-BLED score.

In the LAAEV < 25 cm/s group, one patient had a pericardial tamponade and required percutaneous drainage, and one developed acute left heart failure within 2 days after the procedure; the second patient’s symptoms improved after the administration of diuretics.

In the LAAEV ≥ 25 cm/s group, one patient developed an inguinal hematoma after compression bandaging, and one developed cerebral hemorrhage at 3 months after surgery; the second patient’s symptoms improved after conservative treatment.

All implants in both groups were well sealed (peri-device leaks < 5 mm). There was no occurrence of device dislocation, thromboembolic events, or ischemic stroke.

## Discussion

Previous research conducted by the authors of the present study showed that the LA function in patients with persistent AF improved 1 year after receiving CA alone and LAAC+CA. It was inferred that the improvement of LA function can be mainly attributed to CA, and that additional LAAC did not affect the improvement of LA function [7]. However, the effect of different LAAEVs on the improvement of LA function after combined surgery has not yet been reported.

The present study found that preoperative LA remodeling was more evident in patients with persistent non-valvular AF and that LA function was worse in the LAAEV < 25 cm/s group than in the LAAEV ≥ 25 cm/s group. It also found that, after combining CA with LAAC to restore sinus rhythm, the left atrium of the patients in both groups underwent reverse remodeling, and LA function was improved. Furthermore, the LAV_max_ and LAV_min_ decreased more significantly in the LAAEV < 25 cm/s group than in the LAAEV ≥ 25 cm/s group, and the LA storage function, conduit function, and pump function increased more significantly in the LAAEV < 25 cm/s group than in the LAAEV ≥ 25 cm/s group.

### Comparison of the structure and function of the left atrium in the two groups before surgery

The LA has different functions during a cardiac cycle [15]. During ventricular systole, the mitral valve closes, and the LA stores the blood returning from the pulmonary veins, thus fulfilling a storage function. In the early period of ventricular diastole, the mitral valve opens, and the blood returning from the pulmonary veins to the LA enters the left ventricle, thus acting as a channel. At the end of ventricular diastole, approximately 25% of the blood is discharged to the left ventricle when the atria contract, thus acting as a pump.

In the present study, the LAV_max_ and LAV_min_ were higher, and the strain index was lower, in the LAAEV < 25 cm/s group than in the LAAEV ≥ 25 cm/s group. These results are consistent with the results of previous studies [14]. There was no difference in LAD identified between the two groups, however. This may be related to the methods used to measure the LA anteroposterior diameter. Due to the influence of the thoracic volume and the LA usually being asymmetrically enlarged, the LA often has an increase in the inner and outer diameters or the upper and lower diameters; hence, this method cannot accurately reflect LA size [16].

The volume of the LA reflects LA function better than its inner diameter. The American Society of Echocardiography recommends using the biplane ellipsoid model or the Simpson method to measure LA volume [17]. Two-dimensional speckle tracking cardiac color Doppler ultrasound is a new method for evaluating LA function; its advantages are that it has high repeatability and accuracy and no angle dependence [18].

### Changes in LA structure and function after combined surgery

Previous studies have confirmed that CA restores the sinus rhythm in patients with persistent AF and reduces LA volume significantly compared with before surgery. Furthermore, LA function has been found to improve after CA, suggesting that CA has a positive effect on LA structure and function [6, 7].

As a part of the LA, the LAA has a specific capacity and is more flexible than the LA structure. When the LA volume or pressure is overloaded, the LAA can expand and become a vital storage chamber; this can relieve stress in the LA and ensure the filling of the left ventricle [8]. The separation of the LAA from the LA, however, leads to the disappearance of the storage and buffering functions of the LAA; this reduces the compliance of the LA, which causes it to suffer an increase in pressure and volume overload [19]. Information on the influence of LAAC on LA structure and function is controversial [9–12], but the present study showed that LAAC+CA can improve LA structure and function. Furthermore, a study on combined surgery found that patients with a sinus arrhythmia undergo significant LA reverse remodeling [20], and a study conducted at the Second Hospital of Hebei Medical University found the same after combined surgery and sinus rhythm recovery in patients with persistent AF. It should be noted that the changes in the structure and function of the LA after combined surgery are mainly caused by CA and that sinus rhythm recovery plays a significant role in the process, while LAAC does not affect the improvement of LA function after CA [7]. However, the present study verified that LA structure and function improve after combined surgery.

Left atrial remodeling can cause LA fibrosis and muscle fiber disorder, which play a significant role in the occurrence and maintenance of arrhythmia [21]. The size of the LA is also a factor in the recurrence of AF [22]. Previous studies have shown that LA remodeling is a predictor of adverse factors, such as new-onset heart failure, exacerbation of previous heart failure, stroke recurrence, cardiovascular disease, and an increase in deaths from various causes [23–25]. Therefore, the changes in LA structure and function after combined surgery need to be given greater attention.

### The effect of LAAEV on the structure and function of the LA after combined surgery

The present study found that LA structure and function in patients with AF with an LAAEV of < 25 cm/s were improved to a greater extent after combined surgery than in patients with an LAAEV of ≥ 25 cm/s. A retrospective study found that the smaller the preoperative LAD, the greater the impact on LA structure after LAAC [26]. Furthermore, one study demonstrated that patients who maintain a sinus rhythm after combined surgery achieve reverse remodeling of LA structure.

A lower LAA volume/LA volume ratio before surgery may result in worse LA structure reverse remodeling [20]. The present study found that LA reverse remodeling was more evident and the LA function worse in the LAAEV < 25 cm/s group than in the LAAEV ≥ 25 cm/s group, which is consistent with the results of previous studies [14].

Interestingly, the present study found that postoperative LA structure and function were more significantly improved in the LAAEV < 25 cm/s group than in the LAAEV ≥ 25 cm/s group. This may be due to the fact that lower LAA function leads to less serious postoperative changes in the compliance of the LAA. The smaller the effect of combined surgery on LA structure and function, the more it can reflect the benefits of radiofrequency ablation. In addition, the slower the LAAEV, the higher the probability of LAA thrombosis and the higher the likelihood of AF-induced thromboembolism [14, 27].

The present study also puts forward the factors that affect LA function after treatment with LAAC+CA. If long-term follow-up confirms a greater reduction in the risk of AF thrombosis in the LAAEV < 25 cm/s group than in the LAAEV ≥ 25 cm/s group and a greater improvement in LA structure and function after combined surgery, it indicates that this surgical approach is a worthwhile and reasonable option for patients with non-valvular AF and low LAAEV.

### Limitations

The main limitation of the present study is its small sample size and the fact that the changes in LA structure and function after LAAC alone were not evaluated. However, there have been no previous studies of the effect of LAAEV on LA structure and function after combined surgery, so it is necessary to further explore the factors that affect the role of the LA after combined surgery.

## Conclusion

In patients with persistent non-valvular AF, the preoperative LA remodeling was more evident, and overall LA function was worse, in the LAAEV < 25 cm/s group than in the LAAEV ≥ 25 cm/s group. After combined LAAC+CA surgery and sinus rhythm restoration, both groups achieved LA reverse remodeling, and their LA function improved. The LAV_max_ and LAV_min_ decreased more significantly in the LAAEV < 25 cm/s group than in the LAAEV ≥ 25 cm/s group, and the LA storage function, conduit function, and pump function increased more considerably in the LAAEV < 25 cm/s group than in the LAAEV ≥ 25 cm/s group.

## Data Availability

All data generated or analysed during this study are included in this article. Further enquiries can be directed to the corresponding author.
